# Unusual Presentation of De Winter’s Sign Due to Bezold-Jarisch Reflex in a Patient With Severe Aortic Valve Stenosis

**DOI:** 10.7759/cureus.61563

**Published:** 2024-06-03

**Authors:** Victor H Molina-Lopez, Diego Ortiz-Mendiguren, Porfirio E Diaz-Rodriguez, Stephanie Ortiz-Troche, Francisco Cordova-Perez, Ismael Ortiz-Cartagena

**Affiliations:** 1 Cardiovascular Medicine, Veterans Affairs Caribbean Healthcare System, San Juan, PRI; 2 Internal Medicine, Veterans Affairs Caribbean Healthcare System, San Juan, PRI; 3 Interventional Cardiology, Veterans Affairs Caribbean Healthcare System, San Juan, PRI; 4 Interventional Cardiology, Pavia Santurce Hospital, San Juan, PRI

**Keywords:** de winter's sign, subendocardial ischemia, de winter ecg pattern, bezold-jarisch reflex, aortic valve stenosis

## Abstract

The de Winter electrocardiogram (ECG) pattern, marked by upsloping ST depression in leads V2-V6, ST elevation in lead aVR, and tall symmetric T waves, typically indicates left anterior descending artery (LAD) occlusion. Traditionally linked to LAD occlusion, it is rare in severe aortic stenosis and the Bezold-Jarisch reflex (BJR). We report an 83-year-old man with severe aortic stenosis who developed hypotension due to bleeding and exhibited the de Winter ECG pattern. This case highlights how severe aortic stenosis and BJR can lead to significant hemodynamic instability and ischemic ECG changes, resolving after hemodynamic stabilization.

## Introduction

The de Winter electrocardiogram (ECG) pattern is a distinctive and critical marker traditionally associated with acute occlusion of the left anterior descending artery (LAD). It is characterized by upsloping ST segment depression in leads V2-V6, ST elevation in lead aVR, and tall symmetric T waves, indicating severe myocardial ischemia [1-3]. Although well-documented in the context of LAD occlusion, its occurrence in other clinical scenarios, particularly those involving complex hemodynamic disturbances, remains underexplored. Severe aortic stenosis poses a unique clinical challenge due to its impact on cardiac output and left ventricular pressure, often leading to subendocardial ischemia and hemodynamic instability. Furthermore, the Bezold-Jarisch reflex (BJR) can exacerbate these conditions by inducing paradoxical bradycardia, vasodilation, and hypotension. The BJR can be observed in patients with profound hypovolemia, myocardial ischemia, aortic stenosis, and coronary reperfusion syndromes [4,5].

This case report describes an unusual presentation of the de Winter ECG pattern in an 83-year-old man with severe aortic stenosis and BJR following post-hemorrhagic hypotension. It highlights the interplay between severe aortic stenosis, BJR, and transient ischemic ECG changes, emphasizing the importance of recognizing the de Winter ECG pattern beyond its traditional association with LAD occlusion.

## Case presentation

An 83-year-old male patient presented with bradycardia, hypotension, diaphoresis, and dizziness eight hours following percutaneous coronary intervention (PCI) via femoral arterial access. The patient was being monitored inpatient after the procedure with cautious hydration for prevention of contrast-induced nephropathy.

His medical history was remarkable for hypertension, insulin-dependent type II diabetes mellitus, stage 4 chronic kidney disease, and smoking history with severe chronic obstructive pulmonary disease. He had been diagnosed with severe aortic valve (AV) stenosis. His medications included glargine basal insulin and Aspart pre-prandial insulin, metoprolol succinate 25 mg daily, losartan 25 mg daily, atorvastatin 80 mg daily, aspirin 81 mg daily, and ticagrelor 90 mg twice daily. The transthoracic echocardiogram (TTE) was remarkable for a peak AV velocity (Vmax) of 5.75 m/s, a mean pressure gradient of 80 mmHg, and an AV area of 0.44 cm^2^. The AV was trileaflet but heavily calcified and sclerotic. The left ventricular ejection fraction (LVEF) was of 55%, with concentric hypertrophy, grade II diastolic dysfunction, and reduced global longitudinal strain. Coronary angiography revealed two-vessel obstructive coronary artery disease with an obstructive lesion of 95% in the mid-segment of the right coronary artery (mid-RCA) due to a heavily calcified ulcerated atherosclerotic plaque, treated with rotablation and a drug-eluting stent that day. Additionally, a hemodynamically significant lesion of 65% was identified in the mid-segment of the left anterior descending artery (mid-LAD) with a hyperemia-free ratio of 0.83 (abnormal if ≤ 0.89). The mid-LAD lesion had been scheduled for a staged intervention. Given his elevated Society of Thoracic Surgeons (STS) 30-Day Predicted Risk of Mortality Score of 10.6%, indicating high surgical risk, he was recommended for transcatheter aortic valve replacement (TAVR) over surgical aortic valve replacement.

**Figure 1:**
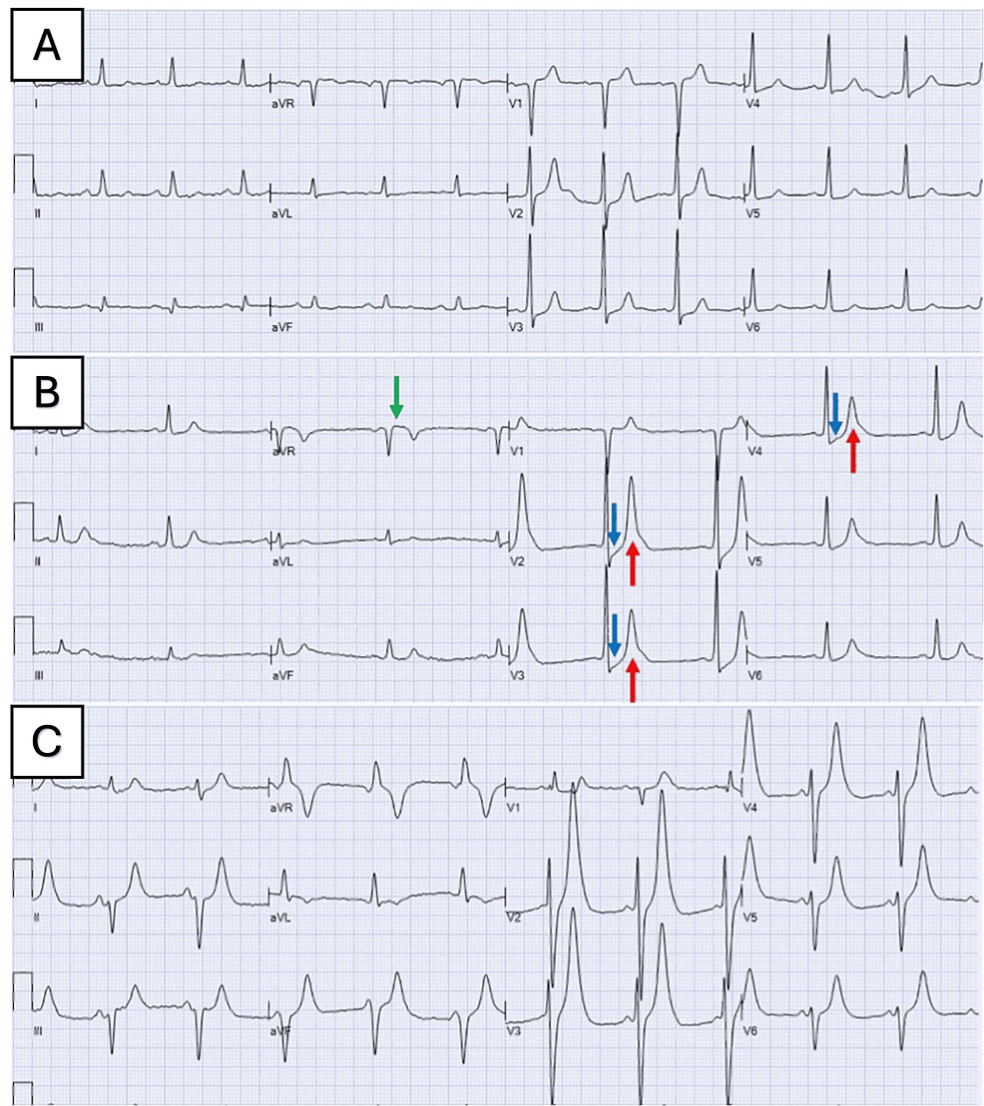
De Winter ECG pattern secondary to Bezold-Jarisch reflex in a patient with severe aortic valve stenosis (A) Baseline ECG. (B-C) ECG demonstrating progression of tall, prominent, symmetrical T waves in the precordial leads (red arrows), with upsloping ST segment depression > 1 mm at the J point in the precordial leads (blue arrows), absence of ST elevation in the precordial leads, and reciprocal ST segment elevation in aVR (green arrow).

Vital signs were remarkable for sinus bradycardia with a rate of 45 beats per minute, blood pressure of 80/50 mmHg, and respiratory rate of 25 breaths per minute. Physical exam was remarkable for a diaphoretic male with pale skin and slow mentation. There was active bleeding in the right groin, originating from the femoral arterial access site for the PCI done earlier that day. The patient received aggressive intravenous hydration and vasopressor support, with sustained pressure applied to the bleeding site. Despite these interventions, his condition deteriorated rapidly, with rapidly escalating doses of norepinephrine despite rapid volume expansion. Quickly after the initiation of norepinephrine, the heart rate increased to over 60 beats per minute. The ECG displayed a progressive emergence of tall, prominent, symmetrical T waves across the precordial leads, ST-segment elevation in aVR, and an upsloping ST segment depression exceeding 1 mm at the J point in V2-V4. Notably, there was an absence of ST elevation in the precordial leads, thus meeting the criteria for the de Winter pattern (Figures 1A-1C).

**Figure 2:**
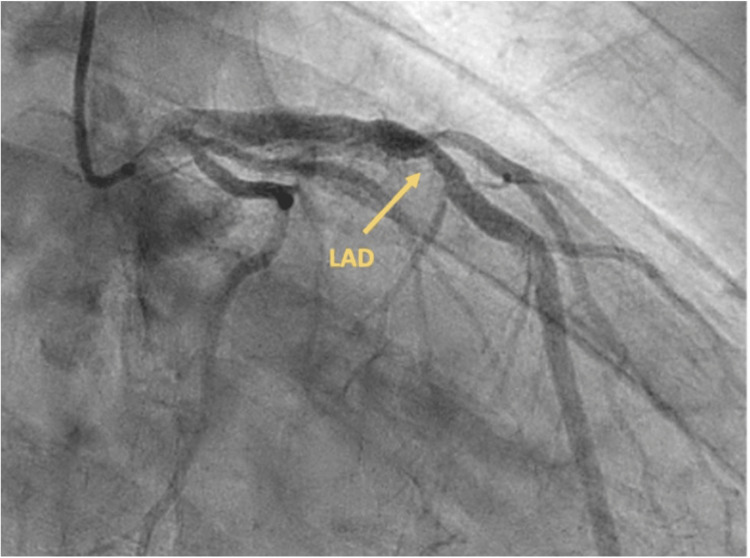
Coronary angiography with mid left anterior descending artery atherosclerotic lesion unchanged from the baseline LAD: Left anterior descending artery

Bedside ultrasound was remarkable for a hypercontractile ventricle and no regional wall motion abnormalities. Point-of-care serum potassium levels and electrolytes were within normal limits. Hemoglobin was 13.1 g/dL. Considering the patient’s worsening condition, he was quickly transferred to the coronary angiography suite for emergency cardiac catheterization. Angiography revealed a patent mid-RCA stent and unchanged obstructive disease in the mid-LAD (Figure 2).

There were no complications involving the aorta or iliofemoral vasculature on peripheral angiography. Soon after the patient’s condition stabilized within two hours from onset, the electrocardiographic abnormalities resolved to his baseline ECG without requiring emergency coronary angioplasty. He was successfully weaned from norepinephrine vasopressor within one hour from the onset of severe hypotension. Lactic acid levels increased mildly to 2.5 mg/dL at two hours from onset. Hemoglobin decreased by 1.5 g/dL over 24 hours of sampling. A computed tomography (CT) scan with intravenous contrast ruled out retroperitoneal bleeding. The patient had an otherwise uneventful clinical course with subsequent intervention of his LAD as scheduled and underwent TAVR with a self-expanding valve. Follow-up evaluation over the next six months demonstrated improved dyspnea and exercise capacity, able to undergo supervised cardiac rehabilitation program successfully. Post-TAVR TTE revealed a mean gradient of 3 mmHg and no perivalvular leak.

## Discussion

The modulation of vasomotor tone involves a complex interplay of various regulatory mechanisms beyond the simple enhancement or reduction of sympathetic activity. The BJR cardio-inhibitory receptors, along with aortic and carotid baroreceptors, play crucial roles in maintaining blood pressure homeostasis. During severe hypovolemia, such as in hemorrhagic conditions, this regulatory interaction can become severely disrupted. The paradoxical activation of the BJR, coupled with baroreceptor failure to maintain dominant control over blood pressure, likely due to impaired afferent sensing or efferent inhibition, results in increased cardioinhibitory receptor firing, leading to bradycardia, vasodilation, and hypotension. Conditions that decrease peripheral vascular resistance and venous return trigger bradycardia as a compensatory mechanism to preserve cardiac filling, highlighting the activation of cardioinhibitory receptors during profound hypovolemia. Additionally, during severe hemorrhage or profound hypovolemia, the relatively empty ventricle can trigger cardiac vagal afferent fibers, eliciting the BJR, which paradoxically results in bradycardia, vasodilation, and hypotension [4,5].

The de Winter ECG pattern, typically characterized by upsloping ST depression at the J point in leads V1 through V6, tall, positive symmetrical T waves, and often ST elevation in lead aVR, has traditionally been associated with LAD occlusions [1-3]. However, this case is unique as no prior reports exist of this pattern in patients experiencing severe transitory shock triggered by the BJR and severe aortic stenosis. The BJR has been observed in patients with profound hypovolemia, myocardial ischemia, aortic stenosis, and coronary reperfusion. In severe aortic stenosis, the fixed cardiac output and inability to effectively increase stroke volume can lead to significant left ventricular pressure and subendocardial ischemia [6]. This condition, combined with vigorous ventricular contractions, can activate cardiac vagal afferent fibers, eliciting the BJR. The reflex can induce paradoxical bradycardia, vasodilation, and hypotension, exacerbating hemodynamic instability. These extreme conditions can significantly alter myocardial perfusion and left ventricular strain, leading to ischemic changes in the ECG, including the de Winter pattern. This case highlights the importance of recognizing a hemodynamically significant lesion in a clinically compromising event. Consequently, in the context of transitory acute circulatory shock induced by the BJR, a hemodynamically significant obstructive lesion, and severe aortic stenosis, the presence of a de Winter ECG pattern may indicate severe subendocardial ischemia related to profound cardiovascular compromise [4,5].

## Conclusions

This case report highlights the importance of recognizing the de Winter ECG pattern beyond its link to LAD occlusion. It describes a patient with severe aortic stenosis where the de Winter pattern appeared due to severe hypotension from bleeding, triggering the BJR. The interplay of fixed cardiac output, increased left ventricular pressure, and strong ventricular contractions in aortic stenosis can activate the BJR, causing hemodynamic instability and ischemic ECG changes. The pattern resolved after hemodynamic stabilization, showing its dynamic nature. Clinicians should be aware of such presentations in severe aortic stenosis and other complex cardiovascular conditions for early identification and management to improve outcomes.
